# Toxins and Antibiotic Resistance in *Staphylococcus aureus* Isolated from a Major Hospital in Lebanon

**DOI:** 10.5402/2011/812049

**Published:** 2011-09-18

**Authors:** Sima Tokajian, Dominik Haddad, Rana Andraos, Fuad Hashwa, George Araj

**Affiliations:** ^1^Genomics and Proteomics Research Laboratory, Department of Biology, Lebanese American University, P.O. Box 36, Byblos, Lebanon; ^2^Department of Pathology and Laboratory Medicine, American University of Beirut Medical Centre, Beirut, Lebanon

## Abstract

Molecular characterization of *Staphylococcus aureus *is of both clinical and infection control importance. Virulence determinants using PCR and multiple drug resistance profiles were studied in 130 *S. aureus* isolates. PCR-RFLP analysis of the 16S–23S DNA spacer region was done to investigate the level of 16S–23S ITS (internal transcribed spacer) polymorphism. Methicillin-resistant *S. aureus* (MRSA), which represented 72% of the studied isolates, showed multiple drug resistance with 18% being resistant to 10–18 of the drugs used compared to a maximum resistance to 9 antibiotics with the methicillin sensitive *S. aureus* (MSSA) isolates. Exfoliative toxin A (ETA) was more prevalent than B (ETB) with virulent determinants being additionally detected in multiple drug-resistant isolates. 16S–23S ITS PCR-RFLP combined with sequencing of the primary product was successful in generating molecular fingerprints of *S. aureus * and could be used for preliminary typing. This is the first study to demonstrate the incidence of virulent genes, ACME, and genetic diversity of *S. aureus* isolates in Lebanon. The data presented here epitomize a starting point defining the major genetic populations of both MRSA and MSSA in Lebanon and provide a basis for clinical epidemiological studies.

## 1. Introduction

*Staphylococcus aureus* are extremely versatile pathogenic bacteria that cause a wide range of syndromes, ranging from minor skin and soft tissue infections to life-threatening pneumonia and toxinoses [1]. The ability of *S. aureus* to cause diseases is multifactorial, a combination of many virulent factors including toxins, secreted exoproteins, and cell surface-associated adhesins [2]. The different virulent factors expressed are controlled by a definite locus named the accessory gene regulator (*agr*) and the staphylococcal accessory regulator (s*ar*) [3]. The exoproteins or exotoxins include haemolysins, different enzymes, and a family of related pyrogenic toxins [4]. These pyrogenic toxins include staphylococcal enterotoxin (SE) A-*↠*E and G-*↠*U subtypes, toxic shock syndrome toxin-1 (TSST-1), and exfoliative toxins (ET) A and B [3]. On the other hand, DNA sequence analysis of the USA 300 clone identified a genetic region and designated the arginine catabolic mobile element (ACME). It was acquired horizontally from *S. epidermidis* or other coagulase-negative staphylococci [4] and inhibited polymorphonuclear cell production. ACME-encoded *arcA *gene encodes for an arginine deiminase pathway and an oligopeptide permease system enhancing colonization, virulence fitness of the strain, and is associated with invasive diseases (necrotizing pneumonia) [5]. 

*S. aureus *resistance against a wide variety of antimicrobials is another important factor and is increasing worldwide, including even resistance to vancomycin, the drug of choice for staphylococcal infections [6]. Strains resistant to penicillin, however, secrete *β*-lactamase (through *blaZ* gene) that hydrolyses the *β*-lactam ring of the antibiotic [7]. Resistance to methicillin is encoded by the staphylococcal cassette chromosome *mec* (SCC*mec*) element, composed of the *mec *gene complex, and the *ccr* (cassette chromosome recombinase) gene complex, encoding for the recombinase gene [8]. SCC*mec *elements have been classified into eight major types (I–VIII), some of which are differentiated further into subtypes. SCC*mec* types I, II, and III, and types IV and V have been associated with hospital-acquired (HA) and community-acquired methicillin resistant *S. aureus *(CA-MRSA), respectively.

Typing is crucial in understanding the epidemiology of pathogens and, hence, the development of public health interventions. Ribotyping is a DNA-based typing method based on the sequencing of the gene coding for rRNAs in bacteria. Genes encoding for rRNA are arranged in an operon in the following order 5′-16S-23S-5S-3′ and is separated by two spacer regions known as the intergenic transcribed spacer (ITS) [9]. The most commonly used components in ribotyping are 16S rRNA gene and the 16S–23S ITS. The ITS has proved to be more variable than the adjacent 16S and 23S ribosomal genes and may allow efficient identification at the species level due to its variability within a genus [10]. This variability is due partly to differences in the number and type of tRNA sequences found within the spacer [11]. The use of sequence polymorphisms and length variations found in the ITS region as a tool for differentiation between closely related organisms is increasing because it can overcome limitations of the resolution of 16S rRNA-based phylogenies [12].

This study aims at characterizing 130 *S. aureus* clinical isolates involved in human diseases based on the determination of antimicrobial resistance profiles, studying the prevalence of exfoliative toxins A (*eta) *and B* (etb), *enterotoxins (SE) A-*↠*E and G-*↠*U, ACME, and TSST-1 and through typing the isolates based on the size of the 16S–23S DNA spacer region and on the patterns obtained by restriction digestion of the amplified spacer region. 

## 2. Materials and Methods

### 2.1. Clinical Isolates

130 *S. aureus* clinical isolates from the American University of Beirut Medical Center (AUB-MC) were used in this study. The isolates were recovered from different sites of infection, age groups, and years. These isolates were identified by different methods including the API and Biolog identification systems and the coagulase test.

### 2.2. Reference Strains

The following *S. aureus* reference strains were used as positive controls: TC-142 (*eta* positive), TC-7 (*etb* positive), and ATCC (BAA-1556D-5) (ACME positive); these strains were thankfully donated by Dr. Michele Bes from the institute of Microbiology, Lyon-France and by Dr. Binh An Diep from the university of California, San Francisco. Reference strains also included the ATCC23235 (*sed* positive), ATCC27664 (*see *positive), NCTC 6571 (*seg* and *sei* positive), NCTC11963 (*tsst* positive), ATCC13565 (*sea *positive), ATCC14458 (*seb *positive), ATCC19095 (*sec *positive), and NCTC10652 (*sej* positive).

### 2.3. Bacterial Storage and Culture Conditions

All clinical isolates were stored in CryoBank tubes (Copan, USA) at both −20°C and −80°C.

### 2.4. Antimicrobial Susceptibility Testing (E-Test)

The antimicrobial resistance of the clinical strains to different antibacterial agents was determined by the standard *E*-test method that comprises a predefined antibiotic gradient strips. Strips were used according to the manufacturer instructions (AB BIODISK, Sweden) to determine the minimum inhibitory concentration (MIC) in *μ*g/mL. Bacterial suspensions were prepared by selecting colonies from overnight cultures on mannitol salt agar plates. The colonies were suspended to sterile tubes containing 5mL of saline solution (0.85% NaCl) to get a suspension with a turbidity similar to the 0.5 McFarland standard. Mueller-Hinton agar plates (Oxoid) were inoculated with the* S. aureus *clinical isolates, and 18 different antibiotics were used including azithromycin (AZ), ciprofloxacin (CI), clarithromycin (CH), clindamycin (CM), chloramphenicol (CL), erythromycin (EM), gentamicin (GM), levofloxacin (LE), nitrofurantoin (NI), norfloxacin (NX), ofloxacin (OF), oxacillin (OX), rifampicin (RI), teicoplanin (TP), tetracycline (TC), trimethoprim (TR), trimethoprim/sulfamethoxazol (TS), and vancomycin (VA). Plates were then incubated for 24 h at 37°C. Following incubation, MIC values that correspond to the edge of the inhibition ellipse intersecting the side of the strips were determined. Resistance or susceptibility profiles were established according to the Clinical and Laboratory Standards Institute [13].

### 2.5. DNA Isolation and Extraction

Bacterial colonies were cultured on TSA plates prior to extraction. Genomic DNA was extracted using QIAamp DNA Mini kit (QIAGEN Inc, Germany) following the manufacturer's instructions.

### 2.6. PCR Amplification

All PCR assays were performed on PerkinElmer GeneAmp 9700 (PerkinElmer, Wellesly, Massachusetts).

### 2.7. PCR for the Detection of eta and etb Encoding Gene

Amplification was performed using the following protocol: An aliquot of 5 *μ*L of DNA (10 ng/*μ*L) was added to 45 *μ*L of reaction mixture containing final concentrations of 1X Ampli*Taq* buffer, 1.5 mM MgCl_2_, 2.5 U of Ampli*Taq* Gold polymerase (Applied Biosystems, Roche), 200 *μ*M each deoxynucleoside triphosphate of either 1 *μ*M of each of *eta*F (5′-CTAGTGCATTTGTTATTCAA-3′) and *eta*R primers (5′-TGCATTGACACCATAGTACT-3′) or 1 *μ*M *etb*F (5′-ACGGCTATATACATTCAATT-3′) and *etb*R primers (5′-TCCATCGATAATATACCTAA-3′) [14, 15]. The amplification was performed with an initial denaturation step for 10 min at 95°C followed by 33 cycles of 94°C for 2 min, 55°C for 1 min, 72°C for 1 min, and a final extension at 72°C for 7 min. PCR products were separated by electrophoresis of 10 *μ*L of reaction product in a 2% agarose gel stained by 0.5 *μ*g/mL ethidium bromide, and visualized using a UV Bioimaging system (GeneSnap system from Syngene).

All PCR assay runs incorporated a negative (one reagent control without template DNA) and a positive control (reference strain used for the gene amplified). Product size was determined by comparison with a 100 bp molecular weight marker (Fermentas, Vilnius, Lithuania).

### 2.8. PCR for the Detection of ACME

Amplification was performed using the following protocol: An aliquot of 2 *μ*L of DNA was added to 20 *μ*L of reaction mixture containing final concentrations of 1X Ampli*Taq* buffer, 2.5 mM MgCl_2_, 0.1 U of Ampli*Taq* Gold polymerase (all from Applied Biosystems, Roche), 0.2 mM each deoxynucleoside triphosphate, the junction between ACME and *orfX *was amplified using 0.4 *μ*M of the *orfX-*specific PCR primer Xsau325 (5′-GGATCAAACGGCCTGCACA-3′), and 0.4 *μ*M AcmeR primer (5′-CCTCCTTCACTTAGCACTG-3′) [16]. The amplification was performed with an initial denaturation step for 12 min at 95°C followed by 30 cycles of 94°C for 30 sec, 61°C for 30 sec, 72°C for 1 min, and a final extension at 72°C for 10 min. PCR products were separated by electrophoresis of 10 *μ*L of reaction product in a 1.5% agarose gel stained by 0.5 *μ*g/mL ethidium bromide, and visualized using a UV Bioimaging system (GeneSnap system from Syngene).

### 2.9. Detection of Enterotoxins and tst Encoding Genes

Multiplex PCR assays were used to detect nine staphylococcal enterotoxin (SE) genes *sea, seb, sec, sed, see, seg, seh, sei, sej, *and the *tst* gene [17]. Two reaction mixtures were used, primers for *sed, see, seg, sei, and tsst * were combined in reaction mixture 1, and primers for *sea, seb-sec, sec, she, and sej *were combined in reaction mixture 2. Amplification was performed using 5 *μ*L of DNA (10 ng/*μ*L) added to 45 *μ*L of reaction mixture containing final concentrations of 1X Ampli*Taq* buffer, 4 mM MgCl_2_, 2 U of Ampli*Taq* gold polymerase (Applied Biosystems, Roche), 400 *μ*M each deoxynucleoside triphosphate, 300 nM of each exotoxin primer set. The amplification was performed with an initial denaturation step for 10 min at 95°C followed by 15 cycles of 95°C for 1 min, 68°C for 45 s, 72°C for 1 min, 20 cycles of 95°C for 1 min, 64°C for 45 s, 72°C for 1 min, and a final extension at 72°C for 10 min. PCR products were separated by electrophoresis of 10 *μ*L of reaction product in a 2.5% agarose gel stained by 0.5 *μ*g/mL ethidium bromide, at 100 V for 100 min, and visualized using a UV Bioimaging system (DigiDoc-IT system, version 2.2.0, 2003).

### 2.10. 16S–23S ITS rRNA Gene PCR Amplification

The 16S***–***23S ITS region was amplified using the following protocol: an aliquot of 2 *μ*L of DNA was added to 20 *μ*L of reaction mixture containing final concentrations of 1X Ampli*Taq* buffer, 2.5 mM MgCl_2_, 0.1 U of Ampli*Taq* gold polymerase (Applied Biosystems, Roche), 0.2 mM each deoxynucleoside triphosphate, 0.4 *μ*M staph ITS-F primer (5′-AGAGTTTGATCCTGGCTCAG-3′), 0.4 *μ*M staph ITS-R primer (5′-CAAGGCATCCACCGT-3′) [18]. The amplification was performed with an initial denaturation step for 12 min at 95°C followed by 30 cycles of 94°C for 30 sec, 42°C for 30 sec, 72°C for 1 min, and a final extension at 72°C for 10 min. PCR products were separated by electrophoresis of 10 *μ*L of reaction product in a 1.5% agarose gel stained with 0.5 *μ*g/mL ethidium bromide, and visualized using a UV Bioimaging system (GeneSnap system from Syngene).

### 2.11. 16S–23S ITS Restriction Digestion

The amplified 16S–23S ITS rRNA gene fragments were digested with 10 U *Taq*I (MBI Fermentas) restriction enzyme. 1 *μ*L of the enzyme was added to 12 *μ*L of the ITS PCR products, 16 *μ*L water, along with 2 *μ*L 10x *Taq*I buffer for 3 h at 65°C (Fermentas) [18]. Products were resolved on a 2% agarose gels in 1x tris-borate-EDTA buffer and were subsequently visualized by UV illumination after ethidium bromide staining.

### 2.12. 16S–23S ITS Gel Extraction

 Gel extraction of the 16S–23S DNA spacer region primary product (1800 bp) was performed using QIAquick Gel Extraction kit (QIAGEN Inc, Germany) following the manufacturer's instructions.

### 2.13. 16S–23S ITS Sequence Analysis

A 500 bp of the purified DNA was subjected to direct sequencing using the Big Dye Terminator Kit (ABI 3130xI Genetic Analyzer, Applied Biosystems). Sequencing was performed in separate tubes, and each reaction (10 *μ*L) consisted of 5 *μ*L of purified DNA and 4 *μ*L of Terminator Ready Reaction mix and was amplified using 2 pmol of the staph ITS-F (5′-AGA GTT TGA TCC TGG CTC AG-3′) and staph ITS-R (5′-CAAGGCATCCACCGT-3′) primers in separate reactions [18]. Sequence homology and identity comparison to other sequences was performed with the NCBI-BLAST sequence search. Phylogenetic tree was constructed by the neighbor–joining method using the CLC Main Workbench version.

## 3. Results

### 3.1. Exfoliative Toxin-Encoding Genes (eta and etb) and ACME

The exfoliative toxin genes were randomly distributed between the 93 MRSA and 37 MSSA. The prevalence of *eta* within both groups was 11% while it was 3% for *etb* in MRSA and 9% in MSSA. ACME was detected in one MSSA isolate (Table 1). The *eta *gene (*n* = 14; 10.8%) was more prevalent than *etb *(*n* = 6; 4.61%), and only three (2%) of the tested isolates harbored both genes. The sizes of amplicons obtained for the exotoxin genes were 119 and 200 bp for *eta* and *etb* genes, respectively, and 333 bp for the ACME, being same as those obtained for the reference strains TC-7 (for *eta*), TC-142 (for *etb*), and BAA-1556D-5 (for ACME). Majority of the isolates, which were previously typed by Tokajian et al. [19] carrying the *eta* gene, belonged to *spa* CC044 (21%) and *spa* CC008 (21%) while the *etb*-positive isolates were detected mainly in CCe *spa*-clonal complex (33%) and the ACME-positive isolate was of *spa* CCd type (Table 1).

### 3.2. Prevalence of Enterotoxins

Isolates were also tested for the presence of nine enterotoxin genes in addition to the TSST-1 gene. The percentage of isolates harboring each of the enterotoxins is shown in Table 2. The nine enterotoxin genes were randomly distributed among the MSSA and the MRSA with the number of toxins detected/isolate ranging between 0 and 4 (Table 3). The gene for the *tsst* was detected in 7 isolates with all being MSSA. Most of the isolates having more than one enterotoxin (4 genes) were resistant on average to only one antibiotic.

### 3.3. Resistance to Antibiotics

**Figure 1:**
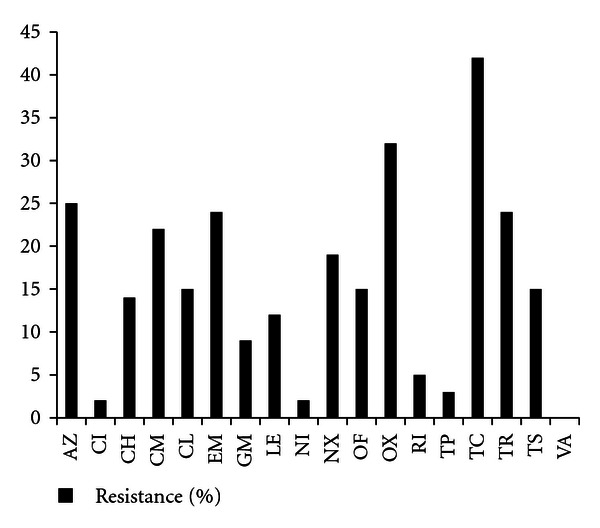
The percentage of resistant isolates to each of the tested antibiotics. The eighteen antimicrobial agents used were azithromycin (AZ), ciprofloxacin (CI), clarithromycin (CH), clindamycin (CM), chloramphenicol (CL), erythromycin (EM), gentamicin (GM), levofloxacin (LE), nitrofurantoin (NI), norfloxacin (NX), ofloxacin (OF), oxacillin (OX), rifampicin (RI), teicoplanin (TP), tetracycline (TC), trimethoprim (TR), trimethoprim/sulfamethoxazol (TS), and vancomycin (VA).

The percentage of resistant isolates to each of the tested antibiotics is shown in Figure 1. All isolates tested showed no resistance to vancomycin, 2% to chloramphenicol and only one showed resistance to nitrofurantoin and teicoplanin. The highest percentage of resistance was to oxacillin (32%; *n* = 41) and tetracycline (42%; *n* = 54). Resistance to other antibiotics was intermediate, ranging between 7% to rifampicin and 25% (*n* = 33) to azithromycin. Multiple drug resistance was detected in both MSSA and MRSA with the maximum number being to 14 drugs (Table 3). Resistance to 10–14 drugs was mainly detected in MRSA *mec* subtypes II and III except for one isolate being resistant to 13 and having* mec* subtype IV.

### 3.4. 16S–23S ITS rRNA Gene PCR Amplification

**Figure 2:**
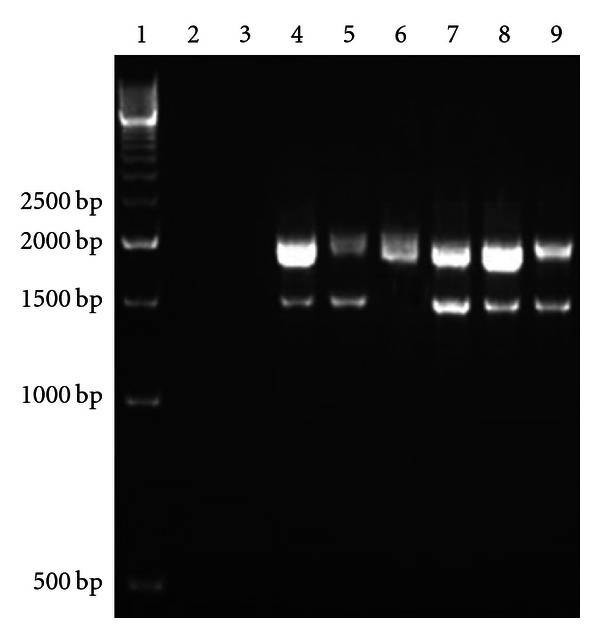
Gel electrophoresis showing 16S–23S DNA spacer region (ITS) amplification products. Lane 1: 500 bp DNA ladder; lanes 2, and 3: negative control (water); lanes 4, 5, 7, 8, and 9: 16S–23S (ITS) positive isolates that belong to ribotype I (1500 bp and 1800 bp); lane 6*: *16S–23S (ITS) positive isolate that belong to ribotype II (1800 bp).

The 16S-ITS rRNA gene was amplified in 15 *S. aureus* isolates representing the major *spa* types. PCR-mediated amplification of this region revealed the presence of two ribotypes. Almost 87% of the isolates belonged to ribotype I with two amplification products size of which were 1500 and 1800 bp. Ribotype II represented 13% of the isolates and gave one band with a size of 1800 bp (Figure 2). All the isolates gave an intense band at 1800 bp, which was designated as the primary product, and most had an additional band, which appeared weaker and was designated as secondary product.

**Figure 3:**
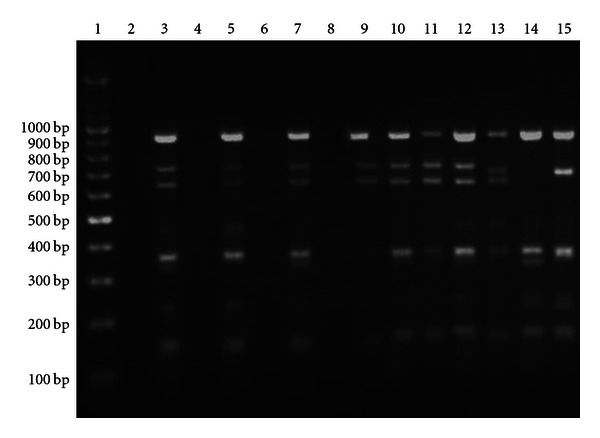
Restriction digestion patterns of the 16S–23S internal transcribed spacer (ITS) amplicons with *Taq*I. Lane1: 100 bp ladder; lane 2: negative control (water); lanes 3, 5, 7, 10, 11, 12, 17, 18, 19, and 20: ITS restriction products for pattern I; lane 9: ITS restriction products for pattern II; lane 13: ITS restriction products for pattern III; lane 15: ITS restriction products for pattern IV; lanes 14 and 16: ITS restriction products for pattern V.

16S–23S ITS-amplified products were digested using *Taq*I restriction enzyme (Table 4 and Figure 3). Electrophoresis and fragment size analysis of restriction products revealed the presence of five banding patterns [four patterns in Ribotype I (lanes 3, 5, 7, and 13) and only one in Ribotype II (lanes 14 and 16)], but the difference only between Ribotype I and II was significant enough totaling to three bands (Figure 3).

### 3.5. Phylogenetic Analysis

To evaluate the phylogenetic relationships between the 15 *S. aureus *representative isolates, we extracted and partially sequenced the 16S–23S ITS rDNA primary product (1800 bp) that was detected in all. Two main clusters were detected and designated as A and B. The first contained S78 (MSSA* spa* t1149) and S89 (MSSA *spa *t937) isolates, and the second contained S1 (MRSA-IV *spa* t044), S3 (MRSA-II *spa *t002), S12 (MSSA *spa* t008), S19 (MRSA-IV *spa* t068), S34 (MRSA-V *spa* t267), S38 (MRSA-III *spa* t044), S40 (MRSA-IV *spa* t537), S75 (MSSA *spa* t021), S88 (MSSA t055), S111 (MRSA-IV *spa* t4098), S113 (MRSA-IV *spa* t279), S121 (MRSA-IV *spa* t660), and S130 (MRSA-IV *spa* t044) isolates. In cluster A both strains were closely related. Sequencing of the primary product revealed that Ribotype I representatives belonged to cluster A, while all other isolates representing Ribotype II were in cluster B. Additionally strains showing similar restriction patterns were clustered together (S1, S34, S38, and S40 all had the same restriction pattern the same applies for S78 and S89).

## 4. Discussion

*S. aureus* is one of the most common causes of CA- and HA-localized and -systemic infections. Nearly all isolates of *S. aureus* produce enzymes and a range of more than 30 different extracellular proteins, most of which play a direct role in pathogenesis [20]. This study aimed at defining the prevalence of virulent determinants (exfoliative toxins, enterotoxins, TSST-1, and ACME), drug resistance and typing of a diverse set of 130 *S. aureus* clinical isolates. The *eta *gene (11%; *n* = 14) was more frequently detected than the *etb *gene (5%; *n* = 6), and only 2.3% (*n* = 3) of all isolates tested harbored both genes. Landhani et al. [20] in the United Kingdom and Ireland, showed that 32% of *S. aureus *isolates produced ETA and only 12% produced ETB. The higher prevalence of the *eta* gene in staphylococci could be explained by its greater immunogenicity [21]. There are reported geographic variations in the prevalence of different ET isoforms. The majority of previous reports confirmed that ETA was the predominant ET isoform in Europe, North America, and Africa which was similar to our findings, whereas ETB-producing isolates were shown to be more frequent in Japan [20–23]. ACME contributed to the success in disseminating the USA300 isolate through enhancing virulence and colonization of humans. In this study, ACME was detected in only one MSSA isolate that was recovered from a skin infection. Goering et al. [16] however, also revealed that only 2 out of 22 USA300 MSSA isolates from California and Nevada were ACME positive. To date, ACME has been reported predominantly among USA300 isolates harboring specific SCC*mec *types (IVa and II) and MLST clonal complexes (CCs) (CC8 and CC5) genetic background, and it has been detected also in England among *S. aureus* isolates with SCC*mec *type IVa [16, 24]. The three virulent determinants (*eta*, *etb*, and ACME) in this study were variably distributed among 16 different *spa* types belonging to 12 different *spa* CCs, possibly because they are located on prophage, plasmid, and a pathogenicity island, which are mobile vectors of horizontal transfer [24]. 

Moreover, the most prevalent enterotoxin gene among the tested isolates in this study was *sei *(59%), which agreed with the findings of Becker et al. [25] in Germany (55%) while it was the *sea* in Jordan (65%) [26]. Percentage of isolates positive for *seb *was 8, which was again in harmony with the results obtained by Becker et al. [25]. In contrast, 25% of the isolates harbored the *seg* gene compared to only 1.4% in Japan [27]. The gene coding for TSST-1, however, was detected only in 7% of the isolates in this study, which matched with the results of Srinivasan et al. [28] in the United States but was lower than the percentage reported by Becker et al. [25] (20%). It's interesting to note that none of the isolates undertaken in this study had the *see* gene, which was the case with clinical isolates studied in Jordan [26]. On the other hand, isolates harboring the enterotoxin genes were randomly distributed among males and females, age categories, site and date of infection, and type (MSSA versus MRSA). It was notable that *tst*-positive isolates were all MSSA, being recovered from abscesses, pus, sputum, or blood. Finally, isolates harboring toxin genes were not multiple drug resistant, and the opposite was also true. 

Multidrug resistance is very common in *S. aureus*, which is now considered as a leading cause of nosocomial infections [27]. The widespread *S. aureus* isolates that are resistant to many antimicrobial agents such as the semisynthetic penicillins (methicillin, oxacillin, and nafcillin), macrolides, tetracyclines, and aminoglycosides made the treatment of staphylococcal infections a global challenge [28]. In this study, resistance to tetracycline was the highest (48%) with similar result being previously obtained by Hamze et al. [29] with samples collected from north Lebanon (44%). Percentage of resistant strains, however, was higher in Australia (80%) [30], while lower in the United States (5%) [31]. Almost 32% of the isolates undertaken in this study were oxacillin resistant which was significantly lower than the percentage detected in Belgium (almost 99%) by Maskell et al. [32]. Percentage of resistance to azithromycin (25%), trimethoprim (24%), and erythromycin (24%) was almost the same and was in harmony with resistance to azithromycin in the UK (23%) and the United States (26%) [31, 32]; it was lower for azithromycin (12%) in Japan [33] and higher to trimethoprim in the UK (69%) [34] and Australia (82%) [30]. This study also revealed an increase in the percentage of strains resistant to erythromycin (24%) compared to results obtained by Baddour et al. [35] where the percentage of resistant strains were much lower being only 7%. Resistance reported for erythromycin was higher in the UK [34] and Australia [30] being 90% and 89%, respectively, while only 12% in Japan [33]. It's noteworthy that almost 15% of the isolates were resistant to trimethoprim/sulfamethoxazol, which agreed with results obtained by Baddour et al. [35]. A significant increase was also detected in this study with the percentage of isolates resistant to clindamycin (22%) and gentamicin (9%) compared to what was reported previously [29] with only 4% of the strains being resistant to clindamycin and 3% to gentamicin. The percentage of strains resistant to teicoplanin, nitrofurantoin, chloramphenicol, and vancomycin detected in this study was either very low or absent. Similar results were also previously reported for both teicoplanin and vancomycin [34–36] while it was higher to chloramphenicol [34, 35]. Multiple drug resistance was detected in both MSSA and MRSA with the maximum number being to 14 drugs. Resistance to 10–14 drugs was mainly detected in MRSA *mec* subtypes II and III except for one isolate being resistant to 13 and having* mec* subtype IV. These findings confirm along with the *mec* subtyping that the majority of the multiple drug-resistant isolates probably were hospital acquired. 

The 16S–23S internal transcribed spacer (ITS) PCR amplification can be used for typing *S. aureus* strains. This region is known to be extremely variable between species, strains, and even among different operons within the same cell and has been widely used to study evolutionary relationships among closely related microorganisms [37, 38]. Amplification of the 16S–23S DNA spacer region revealed the presence of two ribotypes. Ribotype I gave two amplification products with a size of 1500 and 1800 bp, which was consistent with the findings of Sudagidan et al. [18], while ribotype II gave one intense band at position 1800 bp. Ribotype I was the most frequent accounting for 87% of the isolates, whereas ribotype II represented only 13%. Most of the isolates gave additional weaker band, and the source of this secondary product might be the presence of more than one rRNA operon in those isolates with the upper band (1800 bp) containing one or two tRNAs and the lower band (1500 bp) none or only one tRNA-encoding gene [39, 40]. Couto et al. [39] characterized the spacer sequences of *S. aureus* strains and identified 9 rRNA operons, and reported that 3 of these spacers contained the tRNA^Ile^ gene and 2 contained both the tRNA^Ile^ and the tRNA^Ala^ genes, while the remaining 4 16S–23S spacers have no tRNA gene. It is noteworthy that the observed ITS size variability alone was not enough to subtype *S. aureus* isolates at the strain level, and consequently the amplified 16S–23S DNA spacer region was subjected to restriction digestion using *Taq*I restriction enzyme. Previously, ITS RFLP using *Taq*I has been employed for the identification of bacteria of diverse origin including lactobacilli, thermophilic bacilli [40], and alkalophilic bacilli [41] and was also used by Sudagidan et al. [18] for subtyping 16 staphylococcal species in Turkey. Our results revealed 5 distinct RFLP haplotypes, with those belonging to ribotype I having 4 restriction patterns that matched with the results of Sudagidan et al. [18], while isolates belonging to different ribotypes generated different restriction patterns. This study didn't reveal any correlation between ITS ribotyping and prevalence of the virulent determinants.

Combining 16S–23S ITS PCR-RFLP with partial sequencing of the primary product and the construction of a phylogenetic tree revealed the presence of 2 clusters A and B. Isolates belonging to each cluster harbored the same ITS amplicon sizes and similar restriction patterns. These findings matched with the results of Osorio et al. [42], who reported that the intraspecies evolutionary divergence is due to polymorphisms clustered within the ITS variable region where a number of insertion and deletion events occur. The ITS-based identification method can be used to generate molecular fingerprints of *S. aureus *for characterization of isolates and could have useful applications for evolutionary studies. The major drawback, however, is the absence of sufficient information about rRNA copy number and size, the number of tRNA encoding genes and ITS sequence and size in *S. aureus* isolates.

## 5. Conclusion

To the extent of our knowledge, this is the first study that demonstrates the prevalence of virulent determinants and describes the use of 16S–23S ITS PCR-RFLP and its partial sequencing for the subtyping *S. aureus *isolates in Lebanon. The results show that the virulence gene content of *S. aureus* isolated from Lebanon varies widely. The use of efficient and accurate epidemiological typing methods is a prerequisite for monitoring and for limiting the occurrence and spread of epidemic clones within and between hospitals.

## Figures and Tables

**Table 1:** Characteristics of MRSA and MSSA isolates harboring the ACME, *eta* and *etb* genes (*spa* and *mec* typing retrieved from Tokajian et al. [19]).

No. of isolates	MRSA/MSSA (SCC *mec *subtype)^a^	*spa* type^b^	Toxins (ETA and ETB)^c^
3	MRSA (IVc)	t044	ETA
1	MRSA (IVc)	t032	ETA, ETB
1	MRSA (III)	t021	ETA
1	MRSA (IVc)	t037	ETA
1	MRSA (IVc)	t008	ETA
1	MSSA	t937	ACME
1	MSSA	t1439	ETA
1	MSSA	t2179	ETA
1	MRSA (IVc)	t304	ETA
1	MRSA (II)	t002	ETA
1	MSSA	t729	ETB
1	MRSA (IVc)	t3468	ETB
1	MRSA (IVc)	t537	ETA, ETB
1	MSSA	t159	ETA, ETB
1	MSSA	t209	ETA
1	MSSA	t1515	ETB

^a^ MRSA: methicilin Resistant *Staphylococcus aureus*, MSSA: Methicilin Sensitive *Staphylococcus aureus*, SCC*mec*: staphylococcal cassette *mec*

^b^ *spa*: *Staphylococcus aureus *Protein A

^c^ETA: exfoliative toxin A; ETB: exfoliative toxin B.

**Table 2:** Percentage of isolates harboring the different exotoxin genes.

Enterotoxin genes^a^	% of isolates harboring enterotoxins (number)
*Sea*	25 (*n* = 33)
*Seb*	8 (*n* = 11)
*Sec*	5 (*n* = 6)
*Sed*	11 (*n* = 14)
*See*	0 (*n* = 0)
*Seg*	25 (*n* = 32)
*She*	7 (*n* = 9)
*Sei*	60 (*n* = 77)
*Sej*	1.5 (*n* = 2)
*Tsst*	5 (*n* = 7)

^a^ *Sea*-*j*: staphylococcal Enterotoxin a-j, *Tsst*: toxic shock Syndrome Toxin.

**Table 3:** Characterization of *S. aureus* isolates through the determination of antibiotic resistance profiles and prevalence of virulence factors.

MRSA (93 Isolates)^a^	Number of drugs	*eta *gene^c^	*etb *gene^c^	Enterotoxins and TSST-1^d^
*n* = 59	0–2	7%	3%	0–5%
*n* = 23	3–9	26%	13%	0–13%
*n* = 11	10–14	18%	0	0–27%

MSSA (37 Isolates)^b^	Number of drugs	*eta *gene	*etb *gene	Enterotoxins and TSST-1

*n* = 27	0–2	11%	0%	0–15%
*n* = 12	3–9	8%	8%	0–33%

^a^ MRSA: methicilin resistant *Staphylococcus aureus*.

^b^ MSSA: methicilin sensitive *Staphylococcus aureus*.

^c^ *eta*: exfoliative toxin A), *etb*: exfoliative toxin B.

^d^ TSST-1: toxic shock syndrome toxin-1.

**Table 4:** Restriction patterns of the 16S–23S ITS^a^-amplified products.

Groups	Restriction patterns	Isolates no.	Sizes of the amplification products
I	1	*n* = 10 (S1, 3,12, 34, 38, 40, 111, 113, 121, and 130)	150, 250, 325, 350, 450, 650, 725, 900
	2	*n* = 1 (S19)	150, 350, 650,750, 900
	3	*n* = 1 (S75)	150, 350, 450, 675, 700, 900
	4	*n* = 1 (S88)	170,250, 325, 350, 750, 900

II	5	*n* = 2 (S78, and 89)	150,250,325,350, 900

^a^ ITS: internal transcribed spacer.
